# Draft Genome Sequence of *Leptolinea tardivitalis* YMTK-2, a Mesophilic Anaerobe from the *Chloroflexi* Class *Anaerolineae*

**DOI:** 10.1128/genomeA.01356-15

**Published:** 2015-11-19

**Authors:** Lewis M. Ward, James Hemp, Laura A. Pace, Woodward W. Fischer

**Affiliations:** a Division of Geological and Planetary Sciences, California Institute of Technology, Pasadena, California, USA; b Department of Medicine, University of California San Diego, La Jolla, California, USA

## Abstract

We present the draft genome sequence of *Leptolinea tardivitalis* YMTK-2, a member of the *Chloroflexi* phylum. This organism was initially characterized as a strictly anaerobic nonmotile fermenter; however, genome analysis demonstrates that it encodes for a flagella and might be capable of aerobic respiration.

## GENOME ANNOUNCEMENT

*Leptolinea tardivitalis* YMTK-2 was originally isolated from sludge granules of an upflow anaerobic sludge blanket (UASB) reactor used in wastewater processing (1). Closely related stains have been reported from other anaerobic wastewater treatment systems, soils (2), and aquatic moss pillars from Antarctica (3). *L. tardivitalis* is a filamentous, nonsporulating organism that can ferment a number of sugars and fatty acids (1). It grows optimally at 37°C (range 25 to 50°C) and pH 7.0 (range pH 6.0 to 7.2).

The genome of *Leptolinea tardivitalis* YMTK-2 (DSM 16556) was sequenced as part of a project to expand the phylogenetic breadth of *Chloroflexi* genomes. Genome sequencing was performed at Seqmatic using the Illumina MiSeq sequencing platform. SPAdes version 3.1.1 (4) was used to assemble the genome. The genome was screened for contaminants based on sequence coverage, GC composition, and BLAST hits of conserved single copy genes. Genome annotation was performed using the NCBI Prokaryotic Genome Annotation Pipeline. The draft genome is 3.74 Mb in size, assembled into 15 contigs. It encodes 3,349 genes, 2,939 coding sequences, 1 16S RNA, 46 tRNAs, and 5 CRISPR arrays. It is estimated to be ~96% complete based on conserved single copy genes (107/111).

All *Anaerolineae* strains isolated to date have been classified as strictly anaerobic fermenters (1). However, genome analysis suggests that *L. tardivitalis* has a richer physiology than previously recognized. It encodes for both Complex I (NADH dehydrogenase) and quinol *bd* oxidase (5), suggesting that it might be capable of microaerobic respiration. *L. tardivitalis* is missing genes for LPS biosynthesis and known outer membrane proteins, suggesting that it does not have an outer membrane (6). Furthermore, *L. tardivitalis* encodes for Gram-positive flagella, making it likely that it is motile under certain conditions.

### Nucleotide sequence accession number.

This whole-genome shotgun project has been deposited in DDBJ/EMBL/GenBank under the accession number LGCK00000000.
